# Atrial fibrillation among patients under investigation for suspected obstructive sleep apnea

**DOI:** 10.1371/journal.pone.0171575

**Published:** 2017-02-08

**Authors:** Tijn Hendrikx, Martin Sundqvist, Herbert Sandström, Carin Sahlin, Morteza Rohani, Faris Al-Khalili, Rolf Hörnsten, Anders Blomberg, Per Wester, Mårten Rosenqvist, Karl A. Franklin

**Affiliations:** 1 Public Health and Clinical Medicine, Family Medicine, Umeå University, Umeå, Sweden; 2 Clinical Science and Education, Södersjukhuset, Karolinska Institutet, Stockholm, Sweden; 3 Public Health and Clinical Medicine, Medicine, Umeå University, Umeå, Sweden; 4 Clinical Sciences, Danderyd Hospital, Karolinska Institute and Nordic Heart Center, Stockholm, Sweden; 5 Clinical Sciences, Danderyd Hospital, Karolinska Institutet, and Stockholm Heart Center, Stockholm, Sweden; 6 Surgical and Perioperative Sciences, Clinical Physiology, Umeå University, Umeå, Sweden; 7 Clinical Sciences, Danderyd Hospital, Karolinska Institutet, Stockholm, Sweden; 8 Surgical and Perioperative Sciences, Surgery, Umeå University, Umeå, Sweden; University of Bologna, ITALY

## Abstract

**Study objectives:**

Obstructive sleep apnea is common among patients with atrial fibrillation, but the prevalence and risk factors for atrial fibrillation among patients who are being investigated on suspicion of sleep apnea are not well known. The aim of the study was to estimate the prevalence of atrial fibrillation among patients investigated for suspected obstructive sleep apnea and to identify risk factors for atrial fibrillation among them.

**Methods:**

The prevalence of atrial fibrillation was investigated among 201 patients referred for suspected obstructive sleep apnea. Patients without known atrial fibrillation were investigated with a standard 12-lead ECG at hospital and short intermittent handheld ECG recordings at home, during 14 days.

**Results:**

Atrial fibrillation occurred in 13 of 201 subjects (6.5%), and in 12 of 61 men aged 60 years and older (20%). The prevalence of atrial fibrillation increased with sleep apnea severity (p = 0.038). All patients with atrial fibrillation were men and all had sleep apnea. Age 60 or older, the occurrence of central sleep apnea and diabetes mellitus were independent risk factors for atrial fibrillation after adjustments for body mass index, gender, sleep apnea and cardiovascular disease.

**Conclusions:**

Atrial fibrillation is common among subjects referred for sleep apnea investigation and the prevalence of atrial fibrillation increases with sleep apnea severity. Independent risk factors for atrial fibrillation among patients investigated for suspected obstructive sleep apnea include the occurrence of coexisting central sleep apnea, age 60 years or older and diabetes mellitus.

## Introduction

Obstructive sleep apnea with frequent upper airway obstructions followed by transient hypoxia occurs in as many as 80% of patients with atrial fibrillation and nasal continuous positive airway pressure therapy reduces the recurrence of atrial fibrillation after radiofrequency ablation in these patients [1–4]. Risk factors that are common to obstructive sleep apnea and atrial fibrillation include obesity, male gender, diabetes mellitus and age. Atrial fibrillation occurs in about 4% of subjects in the population aged 60–69 years and in about 10% of subjects aged 70–79 years [5, 6]. Atrial fibrillation is a risk factor for ischemic stroke, systemic embolism, heart failure and premature death [7, 8]. Obstructive sleep apnea is also a risk factor for cardiovascular disease and stroke [9–12]. Gami et al., using a questionnaire, suggested that obstructive sleep apnea was overrepresented among patients with atrial fibrillation [13]. Mehra et al. observed that participants in the sleep heart health study with severe sleep apnea had atrial fibrillation 4-times more often than those without sleep apnea [14]. Both Gami et al. and more recently Cadby et al. followed patients with obstructive sleep apnea prospectively and reported an increased risk for atrial fibrillation [15, 16]. The prevalence of atrial fibrillation, among patients seeking medical attention for sleep apnea investigation is however still unknown.

The aim of the present study was to estimate the prevalence of atrial fibrillation among patients under investigation for suspected obstructive sleep apnea and to identify risk factors for atrial fibrillation among these patients.

## Materials and methods

### Patients

The eligible population comprised 243 consecutive adults referred to the Department of Respiratory Medicine, University Hospital, Umeå, Sweden and to the Stockholm Heart Center, Sweden, for suspected obstructive sleep apnea. Thirty-three patients declined participation. Five patients who did not understand the study information and two who were unable to use a handheld ECG device were excluded. Included were 203 consecutive patients on days when a study nurse was present and handheld ECG devices for atrial fibrillation detection were available from April 2012 until November 2013.

### Baseline investigations

All the patients answered a dichotomous questionnaire about a history of atrial fibrillation, congestive heart failure, hypertension, diabetes mellitus, previous stroke or transient ischemic attack, present medication and smoking. Weight, height and blood pressure were measured and all patients answered the Epworth Sleepiness Scale, which measures daytime sleepiness on eight questions with a score of 0–3 [17].

Overnight ambulatory sleep apnea recordings at home (Embletta X10 Systems, Kanata) included continuous recordings of airflow using a nasal flow pressure sensor, thoracic and abdominal respiratory effort (Xact Trace Belts, ResMed), finger pulsoximetry (Nonin Oximeter XPOD, MI) and a body position sensor.

All recordings were scored manually and the duration of sleep was estimated from the recordings. An obstructive apnea was defined as a drop in airflow of at least 90% of the pre-event baseline for ≥ 10 seconds with continuing abdominal and thoracic movements, according to the American Academy of Sleep Medicine [18, 19]. An obstructive hypopnea was defined as a 30% reduction in airflow for ≥ 10 seconds compared with baseline, accompanied by abdominal and thoracic movements, in combination with an oxygen desaturation of 3% or more. Central apneas were scored at absent respiratory effort throughout the entire period of absent airflow for ≥ 10 seconds. Mixed apneas were scored when both central and obstructive components occurred during an apnea. Sleep apnea was considered when the mean number of apneas and hypopneas per hour of sleep, i.e. the apnea-hypopnea index (AHI), was 5 or more. Obstructive sleep apnea was defined when the obstructive apnea-hypopnea index was 5 or more and central sleep apnea was defined when the central and mixed apnea-hypopnea index was 5 or more. Mild sleep apnea was considered when the apnea-hypopnea index was 5 to < 15, moderate sleep apnea when the apnea-hypopnea index was 15 to < 30 and severe sleep apnea when the apnea-hypopnea index was ≥ 30 [18].

### Outcome measurements

A previous diagnosis of atrial fibrillation was verified from patient records. Patients without previously diagnosed atrial fibrillation were investigated using a standard 12-lead resting ECG. They were also instructed to record a 30-second ECG with a handheld ECG device (Zenicor-EKG®, Sweden) at home, regularly twice a day and when any cardiac symptom was present, for 14 days. The Zenicor-EKG® registers a bipolar extremity ECG lead I from both thumbs for 30 seconds. All the recordings were transmitted to a web-based central database via a built-in SIM card. Atrial fibrillation was defined as irregular supraventricular extra systoles in series for 30 seconds. The handheld ECG device is validated to a 12-lead ECG with a sensitivity of 96% and a specificity of 92% for a correct diagnosis of atrial fibrillation [20]. The Regional Ethics Committee at Stockholm University approved the study protocols and all the participants gave their written informed consent. The study complies with the Declaration of Helsinki.

### Statistical analysis

Continuous variables were presented with the mean and standard deviation (±SD). Categorical variables were presented as numbers and percentages. Binary logistic regression was used for both univariate and multivariate analysis comparing patients with and without atrial fibrillation and variables were presented as the odds ratio (OR) and 95% confidence interval (CI). Covariates were chosen because of their relationship to sleep apnea, atrial fibrillation and stroke. A p-value of < 0.05 was considered significant. SPSS Statistics 22 (IBM Corporation, Route 100, Somers, NY 10589) was used for all calculations. The sample size was estimated at 146 patients plus 22 for potential loss, making 168 patients, to detect a significant difference of p < 0.05 with a power of 80% if the frequency of atrial fibrillation was 5% among patients with sleep apnea and 1.5% in patients without sleep apnea.

## Results

The sleep apnea registration failed in two of 203 included patients, leaving 201 subjects for analysis. They had a mean age of 56 ± 12 years, a mean body-mass index of 30 ± 5.4 kg/m^2^ and 69% were men (Table 1). One hundred and seventy patients (85%) had sleep apnea with an apnea-hypopnea index of ≥ 5 and all but one patient had obstructive sleep apnea. Seventeen patients had central sleep apnea and 16 of these also had obstructive sleep apnea. Thirteen patients had atrial fibrillation, 11 were persistent and two had paroxysmal atrial fibrillation. Ten patients had a previous diagnosis of atrial fibrillation and three were new diagnoses. Among patients with known atrial fibrillation, none had been treated with ablative therapy.

**Table 1:** Baseline characteristics.

Men [n, (%)]	138	(69)
Age [years, mean, SD]	56	±12
Heart Failure [n, (%)]	9	(4.6)
Hypertension [n, (%)]	101	(51)
Diabetes mellitus [n, (%)]	20	(10)
Stroke/Transient Ischemic Attack [n, (%)]	6	(3.1)
Ischemic heart disease [n, (%)]	18	(9.2)
Body mass index [kg/m2, mean, SD]	30	±5.4
Smoking [n, (%)]	16	(8.1)

**Fig 1:**
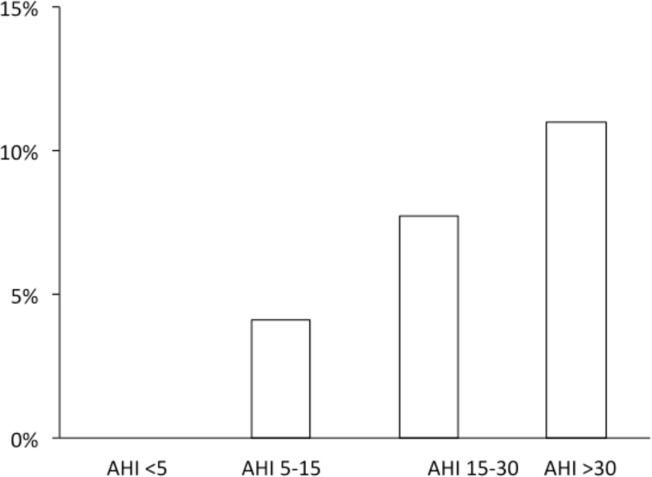
Prevalence of atrial fibrillation in relation to AHI. Prevalence of atrial fibrillation in relation to the severity of sleep apnea measured as the apnea-hypopnea index (AHI). (P = 0.038 for the trend).

Atrial fibrillation occurred in 13 of 201 investigated patients (6.5%), and in 12 of 61 (20%) men aged 60 years and older. The prevalence of atrial fibrillation increased with the severity of sleep apnea, p = 0.038 (Fig 1).

All the patients with atrial fibrillation were men and they all had sleep apnea. Only one of 113 investigated patients younger than 60 had atrial fibrillation (0.9%), and 12 of 88 patients older than 60 had sleep apnea (14%), p <0.000. Atrial fibrillation occurred in six of 17 patients (35%) with concomitant central sleep apnea.

The occurrence of coexisting central sleep apnea, age over 60 years and diabetes mellitus were independently related to atrial fibrillation after adjustments for gender, body-mass index, sleep apnea and cardiovascular disease, including congestive heart failure, hypertension, ischemic heart disease, stroke and previous transient ischemic attacks (Table 2).

**Table 2:** Risk factors for atrial fibrillation.

	Unadjusted OR (95% CI)	p-value	Adjusted OR* (95% CI)	p-value
Central sleep apnea	13.6 (3.89–47.3)	<0.001	27.6 (3.84–199)	0.001
Age ≥ 60 years	17.7 (2.25–139)	0.006	12.2 (1.09–137)	0.043
Body mass index (1 unit)	0.89 (0.78–1.01)	0.077	0.82 (0.67–1.01)	0.059
Cardiovascular disease†	1.85 (0.55–6.22)	0.321	0.26 (0.03–2.08)	0.203
Diabetes mellitus	4.67 (1.29–17)	0.019	22.0 (2.29–211)	0.007

*Gender and sleep apnea (apnea-hypopnea index ≥ 5) were included in the adjusted analysis, but OR and (95% CI) could not be estimated because of convergence failure, as all patients with atrial fibrillation were men and they all had sleep apnea.

†Cardiovascular disease included congestive heart failure, hypertension, ischemic heart disease and previous stroke or transient ischemic attack.

## Discussion

Atrial fibrillation occurred in 7.6% of patients with sleep apnea (13 of 170) among subjects investigated for suspected obstructive sleep apnea. All the patients with atrial fibrillation were men and they all had sleep apnea. Age 60 or older, the occurrence of coexisting central sleep apnea and diabetes mellitus were independent risk factors for atrial fibrillation among those being investigated for a suspicion of sleep apnea. The prevalence of atrial fibrillation also increased with the severity of sleep apnea.

The frequency of sleep apnea among patients with atrial fibrillation has been extensively studied; however, the frequency of atrial fibrillation in sleep apnea has only been investigated in a few previous studies. Leung et al. investigated a subsample of 60 patients with idiopathic central sleep apnea without congestive heart failure, coronary artery disease or a previous stroke and 60 patients with obstructive sleep apnea [21]. They recorded atrial fibrillation during the whole night in 27% of patients with central sleep apnea and in 1.7% of patients with obstructive sleep apnea from a single ECG lead attached to the sleep apnea recordings. Mehra et al. reported that, in the Sleep Heart Health Study, atrial fibrillation occurred in 4.8% of 228 participants with severe disordered breathing versus 0.9% of 338 participants without, p = 0.003 [14]. We investigated a sample of non-selected patients referred for suspected sleep apnea and observed a higher frequency of atrial fibrillation among patients with obstructive sleep apnea. We did not exclude patients with cardiovascular disease and we searched for undiagnosed atrial fibrillation using intermittent recordings with a handheld ECG at home during 14 days, which could explain the higher prevalence of atrial fibrillation in the present study compared with the above-mentioned studies.

We scored apneas and hypopneas manually to distinguish central from obstructive sleep apneas and found that central apneas had a stronger relationship with atrial fibrillation than obstructive apneas. The mechanisms for the association between sleep apnea and atrial fibrillation are, however, still unclear and we can only speculate about them. Obstructive sleep apneas are followed by surges in sympathetic activity and increases in blood pressure and heart rate during apnea [22–24] and previous studies have therefore suggested that obstructive sleep apnea is a risk factor for atrial fibrillation [2, 3, 15]. Central sleep apnea, on the other hand, is generally regarded as the result of congestive heart failure or stroke, because of hypocapnia, reduced cardiac output and enhanced sensitivity to carbon dioxide [25–28]. Atrial fibrillation is highly related to central sleep apnea in heart failure patients and it is suggested that atrial fibrillation is a risk factor for central sleep apnea because it produces a further deterioration in cardiac output [28]. Leung et al. observed a high prevalence of atrial fibrillation also among patients with idiopathic central sleep apnea, i.e. patients without congestive heart failure [21]. They suggested that atrial fibrillation could induce central sleep apnea because of reduced pumping efficiency and raised pulmonary vascular pressure with hyperventilation, hypocapnia and respiratory system instability, or that idiopathic central sleep apnea may predispose some patients to atrial fibrillation because of impaired cardiac electrical stability. A recent study by Ghias et al. supports the hypothesis that central apnea could trigger atrial fibrillation, as they induced atrial fibrillation in anesthetized dogs during atrial and pulmonary vein programmed pacing by turning off a respirator for two minutes [29, 30].

We used a simplified sleep apnea investigation without EEG, which is a limitation when scoring sleep time. Using time in bed as a proxy of sleep time systematically underscores the apnea-hypopnea index. So, in this study, sleep was estimated manually from the respiratory recordings, which is a reliable method compared with polysomnography, with a pooled sensitivity of 93% and specificity of 92% [31]. Another limitation is that thirty-second intermittent ECG recordings could miss some atrial fibrillation episodes compared with continuous ECG recording, thereby underestimating the true prevalence of atrial fibrillation in the studied population.

Oral anticoagulants help to prevent stroke among patients with atrial fibrillation [32–34]. Atrial fibrillation can also be treated with antiarrhythmic drugs and pulmonary vein isolation to restore sinus rhythm. The effect of pulmonary vein isolation is further improved after nocturnal nasal continuous positive airway treatment of concomitant sleep apnea with a 42% relative risk reduction of atrial fibrillation recurrence [4] [35, 36]. Here, we report that atrial fibrillation is common among subjects being investigated for sleep apnea. To prevent stroke, there is evidence not only to search for sleep apnea in patients with atrial fibrillation, but also to search for atrial fibrillation during sleep apnea recordings.

Polysomnography, the golden standard for sleep apnea recordings includes a one-lead ECG (V5), which is often removed in simplified sleep apnea recordings. We suggest that such a one-lead ECG together with an analyzing program should be included also in simplified sleep apnea recordings to detect atrial fibrillation.

## Conclusions

Atrial fibrillation is common among subjects who are referred for sleep apnea investigation and the prevalence of atrial fibrillation increases with sleep apnea severity. Independent risk factors for atrial fibrillation among patients investigated for suspected obstructive sleep apnea include the occurrence of coexisting central sleep apnea, age 60 years or older and diabetes mellitus.

## Supporting information

S1 Dataset Data underlying the findings in the study. (XLSX)
